# Surgery for Liver Metastases From Gastric Cancer: A Meta-Analysis of Observational Studies

**DOI:** 10.1097/MD.0000000000001113

**Published:** 2015-08-07

**Authors:** Luca Martella, Serena Bertozzi, Ambrogio P. Londero, Agostino Steffan, Paolo De Paoli, Giulio Bertola

**Affiliations:** From the Surgical Oncology Department, IRCSS CRO, Aviano, Italy (LM, SB, PDP, GB); SOC of Obstetrics and Gynecology, S. Polo Hospital, Monfalcone, Italy (APL); and Oncological Pathology Unit, IRCSS CRO, Aviano, Italy (AS).

## Abstract

The role of surgical therapy in patients with liver metastases from gastric cancer is still controversial. In this study, we investigated the results obtained with local treatment of hepatic metastases in patients with gastric cancer, by performing a systematic literature review and meta-analysis.

We performed a systematic review and meta-analysis of observational studies published between 1990 and 2014. These works included multiple studies that evaluated the different survival rate among patients who underwent local treatment, such as hepatectomy or radiofrequency ablation, for hepatic metastases derived from primary gastric cancer. The collected studies were evaluated for heterogeneity, publication bias, and quality, and a pooled hazard ratio (HR) was calculated with a confidence interval estimated at 95% (95% CI).

After conducting a thorough research among all published works, 2337 studies were found and after the review process 11 observational studies were included in the analysis. The total amount of patients considered in the survival analysis was 1010. An accurate analysis of all included studies reported a significantly higher survival rate in the group of patients who underwent the most aggressive local treatment for hepatic metastases (HR 0.54, 95% CI 0.46–0.95) as opposed to patients who underwent only palliation or systemic treatment. Furthermore, palliative local treatment of hepatic metastases had a higher survival rate if compared to surgical (without liver surgery) and systemic palliation (HR 0.50, 95% CI 0.26–0.96). Considering the only 3 studies where data from multivariate analyses was available, we found a higher survival rate in the local treatment groups, but the difference was not significant (HR 0.50, 95% CI 0.22–1.15).

Curative and also palliative surgery of liver metastases from gastric cancer may improve patients’ survival. However, further trials are needed in order to better understand the role of surgery in this group of patients.

## Abstract 2

Supplemental Digital Content is available in the text

## INTRODUCTION

Gastric cancer is the fourth most common type of tumor worldwide^1^ and the second cause of cancer-related death worldwide.^1^ Despite the significant reduction of gastric cancer incidence in the last 20 years, we observed an increase in the number of advanced-stage diagnoses.^2^ In Western Europe and in the Anglo-Saxon world, the incidence of hepatic metastases from gastric cancer during the course of the disease varies between 30% and 50%, including both synchronous and metachronous metastases.^3,4^ In particular, at the time of diagnosis 35% of patients present with evidence of distant metastases, and 4% to 14% have metastatic disease to the liver,^5–25^ whereas metachronous metastases after curative gastrectomy are detected in up to 25% to 30% of patients, 80% of which appear within the first 2 postoperative years.

Surgical treatment of hepatic metastases from gastric cancer is currently reason of great debate.^23,26–29^ In fact, although many studies observed no survival difference between patients who underwent liver surgery and those who did not, it appears that in selected cases an aggressive treatment can achieve unexpected results.^16–19,21,24,25,30–36^ Moreover, surgery is not always a viable option, mainly due to multiple hepatic metastases or the presence of extra-hepatic dissemination,^3,4^ and only 0.4% to 1% of metastatic gastric cancer patients result eligible for radical surgery.^5,7,8,37^

In this article, we reviewed the literature and performed a meta-analysis in order to evaluate the survival impact of liver resection in patients with hepatic metastases from gastric cancer.

## MATERIALS AND METHODS

### Search Strategy for Review

A literature search was independently carried out by 3 authors. All information was gathered from Medline, Embase, Ovid, Google Scholar, and Cochrane database for studies published from January 1990 to December 2014 (by online search engines and by JabRef 2.10). Search terms included “liver,” “neoplasm,” “metastasis,” “stomach,” “neoplasm metastasis,” “stomach neoplasms,” “gastric,” “cancer,” and “gastric cancer.” Titles, abstracts, and meta-information resulted from these queries were examined. All articles that referred to the surgical or local treatment of liver metastases from gastric cancer were selected. Full texts were analyzed afterwards. Eventually, bibliographies and citations from full articles and previous review publications were used to identify other additional pertinent articles.

### Inclusion and Exclusion Criteria

All observational and experimental studies that evaluated survival in patients affected by synchronous or metachronous liver metastases from primary gastric cancer and treated with local intraoperative methods were considered. All included studies were observational (level III or IV of evidence, Center for Evidence Based Medicine (CEBM))^38^ and no randomized clinical trial comparing surgical treatment of liver metastases and chemotherapy or medical palliation have been found. We considered, in this meta-analysis, Kaplan–Meier curves or Cox proportional hazards regression models to calculate the survival difference among patients treated with surgical or other local options compared to palliation or systemic treatment. Moreover, we only included articles where a full text was available for data retrieval, performed on human subjects and written in English (we did not contact the authors). We retrieved from full text articles patient treatment time frame, geographic locations, and type of treatment in order to avoid any possible population overlap. In case of 2 or more studies regarding the same set of patients or presenting possible data overlap we selected the 1 with better quality or more detailed data. When discrepancies among the 3 reviewers were found, a joint reevaluation of the original article was performed to address them. Articles written in languages other than English, studies without a control group, or studies about nonhuman subjects were specific exclusion criteria. In addition, letters to the editor without original data, editorials, case reports, and reviews were excluded. Moreover, conference abstracts were excluded due to the lack of details regarding survival data and study design.

### Data Extraction

Three independent reviewers extracted data from the selected articles by using a predefined data extraction form. As previously described, any discrepancies in data extraction or unsuitability for inclusion were discussed^39,40^ and the following information was extracted: authors, year of publication, geographical area, population characteristics (sex, age, etc.), study design, number of patients, type of procedure applied, median follow-up length, surgical morbidity and mortality, hazards ratio (HR) with a 95% confidence interval (95% CI), or HRs extracted from Kaplan–Meier curves. The HR was calculated using methods previously described from data obtained from published reports.^41^ In case, the considered study presented a multivariate analysis we preferred to include the adjusted HR with the relative CI in our analysis.

### Quality Assessment for Included Studies

The quality of each included study was assessed using the Newcastle–Ottawa Scale as previously described.^39,40^ For the purpose of this study we defined as high-quality studies those works that scored 9 or 8 points on the Newcastle–Ottawa Scale, medium-quality studies those that scored 7 or 6 points, and low-quality studies those that scored <6.^39,40^ Discrepancies in quality assessment were solved as previously described.^39,40^

### Data Analysis

Data were analyzed by R (version 3.1.1), considering significant the *P* < 0.05. We calculated a summary statistic considering the HR for survival analysis. Rank correlation test of funnel plot asymmetry was used to test the presence of any publication bias.^42,43^ The I2 index and the Cochran Q were used to assess the heterogeneity among studies. As previously described an I2 index value >50% and a Q statistic *P* value < 0.10 were considered statistically significant signs for heterogeneity.^44^ We applied, where appropriate, the fixed- and random-effect model to calculate the pool estimate. We reported the primary outcome in this meta-analysis as HR (with 95% CI) of overall survival in patients treated with local treatment of gastric cancer hepatic metastases. MOOSE (Meta-analysis Of Observational Studies in Epidemiology) guidelines for accurate performing meta-analysis of observational studies^45^ and PRISMA (Preferred Reporting Items for Systematic Reviews and Meta-Analyses) guidelines checklist^46^ were followed to prepare this meta-analysis. This meta-analysis is exempt from ethical approval as the analysis involves only already published and anonymized data.

## RESULTS

### Search Results

**FIGURE 1:**
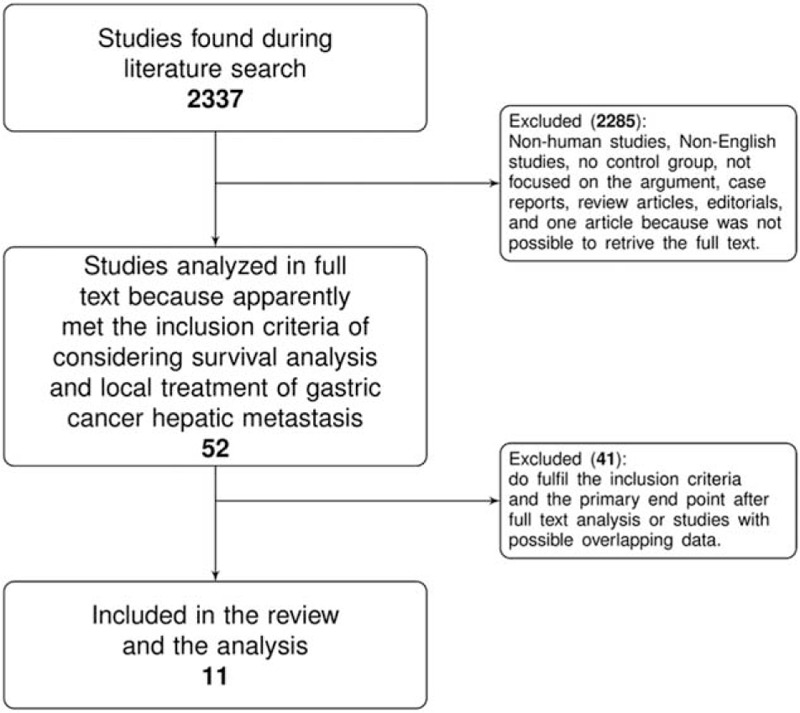
Flowchart of the literature search and selection.

Figure 1 shows the literature search flowchart. During the literature search we found 2337 studies (Figure 1). After reviewing the titles and abstracts we found 2284 articles to be not eligible as they were case reports, review articles, editorials, nonhuman studies or non-English articles, not focusing on the review topic, and others not meeting the inclusion criteria. We identified 53 articles as potentially eligible for this review. However, for 1 article it was not possible to obtain the full text^35^ and for other 41 of these articles either the selected outcome was not described (survival difference between local treatment of gastric cancer hepatic metastases and palliation or systemic treatments), the HR with the relative CI, or Kaplan–Meier curves were not adequately reported. In the Supplemental List 1, http://links.lww.com/MD/A348, we show the included and excluded studies. We finally selected 11 eligible articles (Figure 1).^11,12,31,37,47–53^ All these included 11 research articles were observational studies.

### Characteristics of the Studies

In our meta-analysis, we included 11 observational studies that evaluated the survival rate in patients affected by gastric cancer with hepatic metastasis, comparing curative surgical resection with palliation or systemic therapies. In Table 1, we report the main characteristics of these studies. The total number of patients considered in the survival analysis of the included studies was 1010. The majority of patients in all included studies were males and the median age was around 60 years. The median follow-up time was 13 months (IQR 11–16). Furthermore, the studies were mostly retrospective or retrospective from prospective databases. Among the local treatment of gastric cancer liver metastases we found hepatectomy and radiofrequency ablation (RFA) to be the most frequently used. In 4 of the included studies, it was feasible to assess the prevalence of hepatic metastases in gastric cancer patients.^12,37,50,53^ Two of these studies considered only synchronous gastric cancer liver metastases showing a summary prevalence of 6% (95% CI: 5–7) using a random-effect model,^37,53^ while the other 2 studies considered both synchronous and metachronous gastric cancer liver metastases showing a summary prevalence of 14% (95% CI: 7–27) using a random-effect model.^12,50^ Five of the included studies considered only synchronous gastric cancer liver metastases,^37,47,48,51,53^ other 5 studies considered together synchronous and metachronous gastric cancer liver metastases,^11,12,49,50,52^ while only 1 study considered metachronous gastric cancer liver metastases alone.^31^ As shown in Table 1 hepatectomy was almost always considered in isolated liver metastases and the majority of patients with curative surgery were H1 according to the Japanese Classification of Gastric Carcinoma.^54^ In the 11 included studies, the 5 years overall survival of the best performing local treatment group was ranging between 7% and 60% with a median of 21%. We extracted from the articles the HR and the relative CI, preferring HR corrected by Cox proportional hazards multivariate analysis. In those cases, where the HR was not calculated it was extracted from Kaplan–Meier curves. The excluded studies after the full paper analysis (the second step of our study selection process) are shown in Supplemental List 1, http://links.lww.com/MD/A348; they were all observational studies and survival HR extraction was not feasible.

**TABLE 1:**
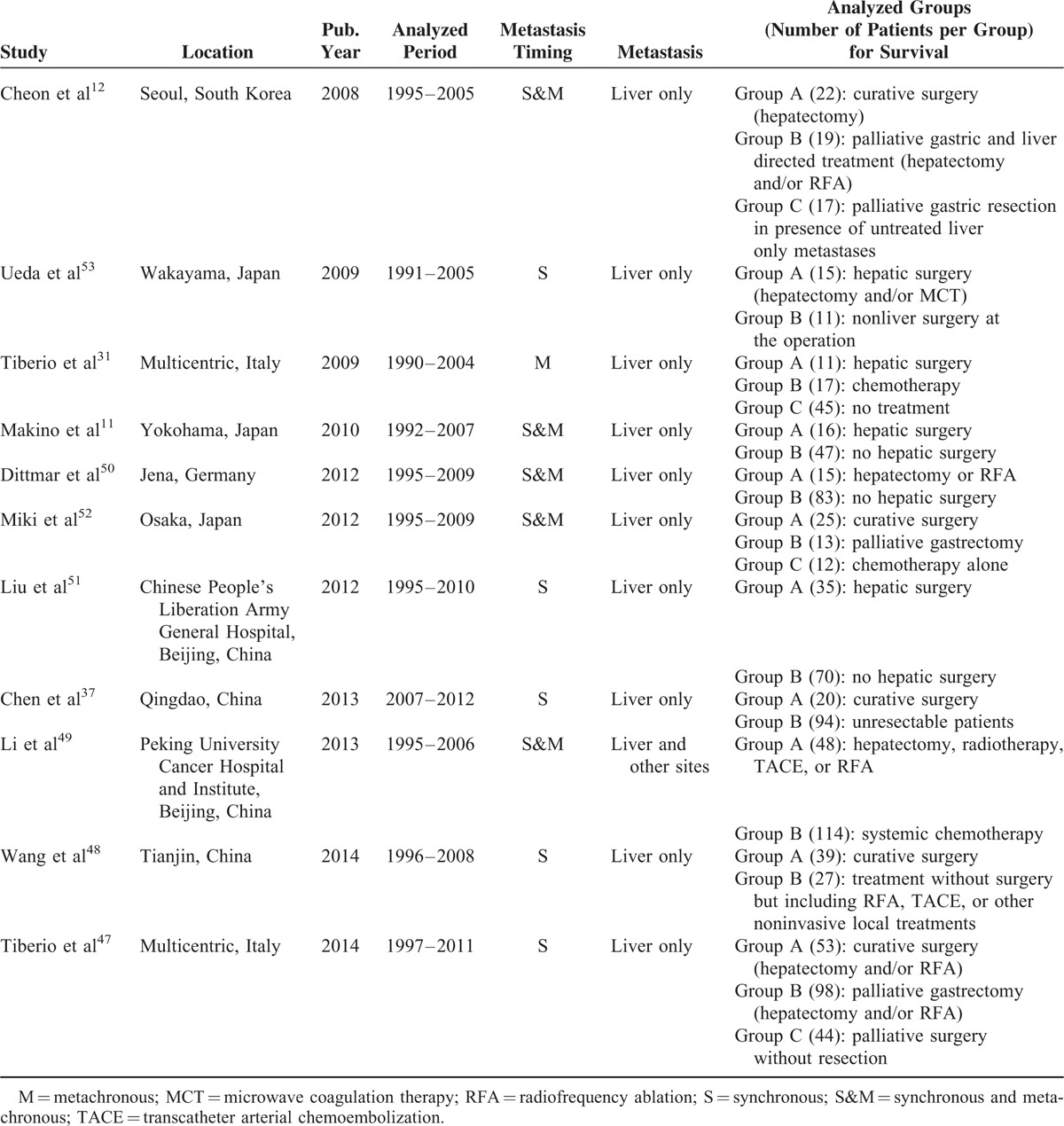
Characteristics of Included Studies

### Quality Assessment of the Included Studies

The quality of the evidence for the role of hepatectomy or other local treatments during surgical procedures for gastric cancer hepatic metastases is low (levels III–IV, CEBM).^38^ However, the studies in our meta-analysis apparently showed an increased survival rate in the groups that underwent local treatment of liver metastases (eg, hepatectomy), in particular in the groups treated by curative hepatectomy and gastrectomy, but none of the included studies was randomized. According to the Newcastle–Ottawa scale for quality median score of the included studies was 7 (IQR 7–8). Five studies were graded 9 or 8 points according to the Newcastle–Ottawa scale for quality (high quality), and 6 studies were graded 7 or 6 (medium quality).

### Main Analysis

**FIGURE 2:**
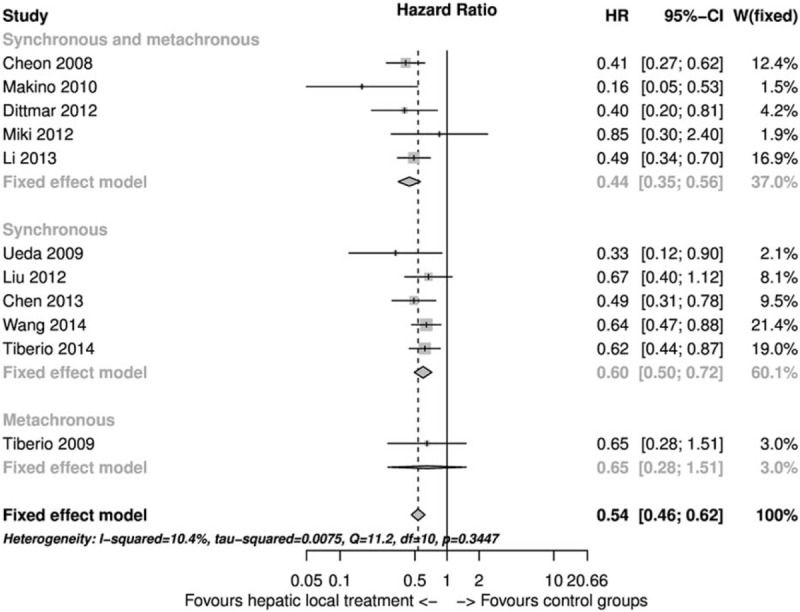
Forest plot of overall survival comparison between gastric cancer hepatic metastasis local treatment versus palliation or systemic treatment.

**FIGURE 3:**
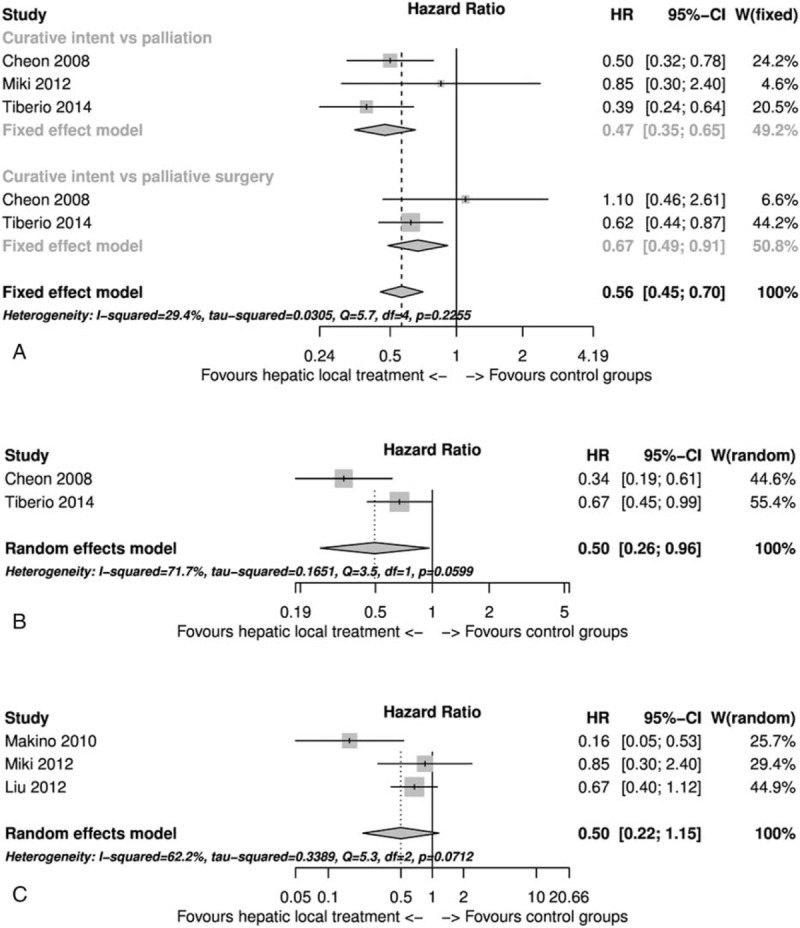
Forest plots. (A) Curative surgery vs surgical or systematic palliation (this strata had no significant heterogeneity) and curative surgery vs palliative surgery with local hepatic treatment (this strata had no significant heterogeneity). (B) Palliative surgery with local hepatic treatment vs surgical or systematic palliation. (C) HR and 95% CI from multivariate analysis of curative surgery vs palliation. CI = confidence interval; HR = hazard ratio.

The 11 selected studies were used to perform the meta-analysis (Figure 2). In Figure 2, the I2 index value was 10.4% and the Q statistic *P* value was 0.345; therefore, we found no heterogeneity among the included studies and we used the fixed-effect model to calculate the pooled estimate. In some of the included studies shown in Table 1 more than 2 groups of patients were analyzed. For the analysis shown in Figure 2, in case of Cheon et al we took into consideration group A and group B together versus group C; in case of Tiberio et al^31^, Miki et al^52^, and Tiberio et al^47^ we considered group A versus B (Table 1). Therefore, considering all the studies together a statistically significant higher survival rate was found in the group of patients treated with local hepatic treatment of gastric cancer metastases HR 0.54 (95% CI: 0.46–0.95) compared to patients who underwent only palliation or systemic treatments (Figure 2). We further analyzed the data concentrating on the timing of metastases and we found a survival advantage in the local treatment of hepatic metastases (Figure 2). Furthermore, in Figure 3A, we show that curative surgery with complete resection of gastric cancer and hepatic metastases had a higher survival rate in comparison to palliative surgery of hepatic metastases or palliation. In Figure 3B, we show that palliative local treatment of hepatic metastasis had a significant survival improvement in comparison to palliation HR 0.50 (95% CI: 0.26–0.96). In Figure 3C, we considered only the studies in which it was possible to extract data from the corrected multivariate Cox regression (heterogeneity was present and a random-effect model was used). In this case as well we found a survival advantage of gastric cancer hepatic metastases addressed with local treatment, but the difference was not significant HR 0.50 (95% CI: 0.22–1.15) (Figure 3C).

### Risk of Bias Assessment

The majority of included observational studies were classified as medium quality. The selection bias was the main limit of the included observational retrospective studies. In fact, different authors have used different types of control groups and had considered in the treatment group a different range of gastric cancer hepatic metastases extension. Of the 11 studies, 6 included claimed a multivariate analysis of factors possibly influencing survival.^11,12,31,49,51,52^ However, in only 3 studies it was possible to extract the multivariate HR and the relative CI in order to perform a summary statistic.^11,51,52^ The majority of the studies with a multivariate analysis found a significant survival improvement with local treatment of hepatic metastases^11,12,31,49^ and in only 2 studies the improvement was not significant after multivariate adjustment.^51,52^ In addition, among the 3 studies included in the meta-analysis of the multivariate HR 2 of them presented a nonsignificant survival improvement after multivariate adjustment.^51,52^ Cheon et al^12^ corrected in multivariate analysis for gastric cancer hepatic metastases extension, timing, sex, and age. In general, it seems that Makino et al^11^ corrected their data for gastric cancer hepatic metastases extension, operability criteria, stage, curability, and chemotherapy (but their application of this process in multivariate analysis was not always clear). Tiberio et al^31^ considered metachronous metastases corrected for gastric cancer hepatic metastases extension, staging, grading, and disease-free survival. Miki et al^52^ conducted a multivariate analysis corrected for gastric cancer stage and multiple hepatic metastases. Liu et al^51^ performed as multivariate analysis corrected for gastric cancer hepatic metastases extension, extent of lymphadenectomy, resection margin, stage, and lymphovascular invasion. Li et al^49^ in their multivariate analysis corrected for previous gastrectomy, extra-hepatic metastases, number of liver metastases, and chemotherapy. Also Tiberio et al^47^ analyzed synchronous liver metastases considering in their analysis hepatic metastases extension, T stage, and chemotherapy. The other included studies did not extensively consider possible confounding factors in their analysis.^37,48,50,53^ Furthermore, surgical morbidity and mortality is not adequately reported suggesting a possible selection bias that excluded severe surgical complications. However, Tiberio et al^47^ found a nonsignificant difference in morbidity among the studied groups and only a significant increase in mortality in patients treated with palliative gastrectomy without local treatment of liver metastases. In general, they concluded that liver local treatment in addition to gastrectomy does not affect operative results, considering as cornerstone the preservation of postoperative liver function.^47^

### Publication Bias

**FIGURE 4:**
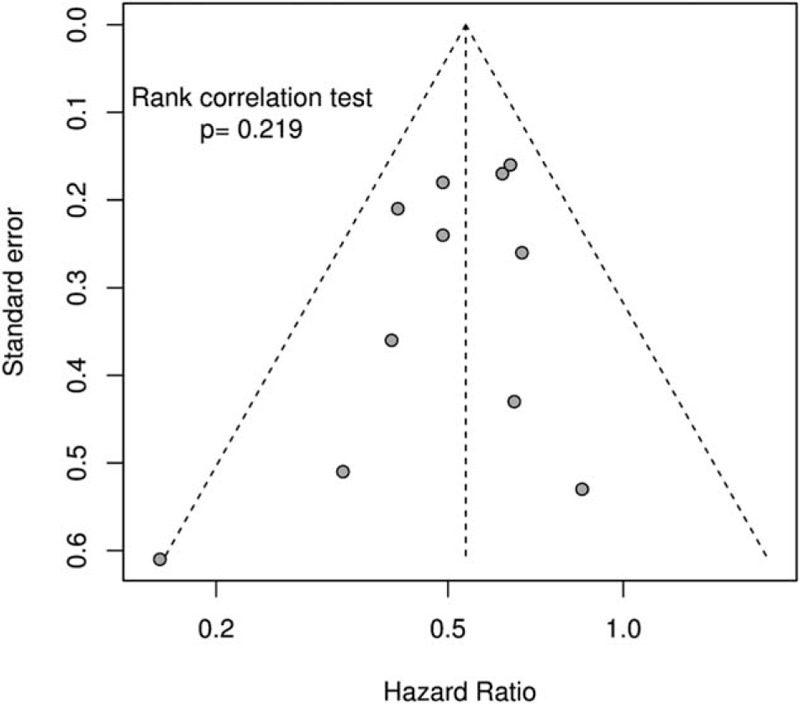
Funnel plot.

The presence of a possible publication bias was examined by a funnel plot (Figure 4). We found no significant publication bias and the rank correlation test of funnel plot asymmetry had a *P* value of 0.219.

## DISCUSSION

After examining all included studies our analysis found a significant survival improvement in patients who underwent local treatment of hepatic metastases compared to those who received palliation or systemic treatment. However, when considering only the studies where it was possible to extract adjusted HR with its relative CI this improvement in survival was present but not significant. Furthermore, we also found palliative local treatment of hepatic metastases to have a significant survival improvement in comparison to palliation without local treatment of hepatic metastases.

The main limitation of this meta-analysis was the presence in literature of observational studies only (cohort or case–control studies) that are at high risk of patient selection bias. Furthermore, it was possible to extract adjusted HR and CI only for 3 studies out of 6 that claimed a multivariate analysis. In fact, in case of observational studies the adjusted HR and CI are of paramount interest for possible confounding factors. Moreover, despite the possibility to extract adjusted HR and CI only from unfavorable studies we still found a survival benefit in the local treatment of hepatic metastases, even if not significant. In addition, we should consider the possible existence of a selection bias in this summary statistic of adjusted HRs due to lack of data presented in the published articles. For this reason, in future observational studies, it is important for the authors to present a multivariate survival analysis reporting at least adjusted HR and CI.

Among the excluded studies, the article whose full text could not be obtained seems to confirm from the abstract's data the findings of the current meta-analysis.^35^ Furthermore, searching trough published works in literature we only found 1 article that consider local re-treatment of liver metastases after recurrence, and the authors found a survival advantage in local treatment repetition.^55^

Resection of liver metastases from gastric cancer was initially indicated in patients with synchronous metastases who had no peritoneal dissemination or other distant metastases and in patients with metachronous metastases without any other detectable lesion,^19^ only if a complete resection of the metastases could be achieved without compromising postoperative liver function.^21^ Thereafter, Roh et al^15^ supported surgery indication in case of single-lobe liver metastases without peritoneal dissemination, hilar node metastases, or distant metastases. Recently, in accordance with the latest findings, the Japanese Gastric Cancer Association revisited its treatment guidelines which, in case of stage IV gastric cancer, recommended only chemotherapy, radiation, palliative surgery, and best supportive care,^54^ in favor of surgical treatment with curative intent for potentially resectable M1 disease, including patients with resectable hepatic metastasis, positive cytological examination of peritoneal washes, or swollen nodes in the para-aortic region.^56^

Unfortunately, in the review of current literature hepatectomy was indicated in only 0.4% to 1% of gastric cancer patients with liver metastases, because most hepatic metastases from gastric adenocarcinoma are multiple, bilateral, and combined with peritoneal or lymph nodes metastases, which directly invade adjacent organs, so that eventually very few patients result good candidates for liver surgery.^57^ Moreover, surgical indications for liver metastases of gastric origin must be carefully determined because of the biological, clinical, and pathological aggressiveness of the disease.^58,59^ However, even if the percentage of patients who may benefit from resection is probably small, our meta-analysis agrees that the best survival rates are associated with surgical treatment, which should be chosen whenever possible.^7^ Moreover, the current evidence (survival advantage of local palliation of liver metastases) suggested the need of additional studies in order to try and widen the indications of local treatment or palliation of hepatic metastases. In addition, the overall 5-year survival rate of metastatic gastric cancer ranges between 0% and 10%,^9,10,60^ whereas it rises up to 20% after curative hepatectomy according to literature^15–19,24,61^ and also the 11 studies included in this meta-analysis (median 5-year overall survival 21%).

The prevalence of synchronous liver metastases in the studies included in this meta-analysis (6%) was similar to the findings of other published studies (4–14%) while the prevalence of synchronous and metachronous dissemination was lower (14% vs 30–50%) probably because these patients had previously been included in surgical studies and were therefore already selected.^5–25,37,50,53^

In current literature, many factors seem to influence the survival rate of gastric cancer patients with hepatic metastases. In particular, the prognosis seems to be significantly worsened by multiple factors: a greater extent of hepatic involvement (H3) or macroscopic peritoneal dissemination (P1) detected at surgical exploration, a greater number (>1) and size of hepatic metastases in H1 to H2 and P0 patients,^12,13,53,62^ a greater tumor size (T4), nodal involvement (N+ independently from the extension of the metastatic spread) or higher tumor grading (G3),^14,31,63^ and the diagnostic timing of liver metastases (metachronous metastases correlate with a poorer prognosis).^19,21,59^ Therefore, these factors should be considered as possible confounding elements in future studies. In addition, considering all these prognostic factors, some authors suggested the necessity to clearly identify which patients could benefit from a surgical approach, in order to offer a better chance of treatment to those who present with good prognostic factors and to avoid overtreatment of the others.^7^

Taking into account local procedures for hepatic metastases, no consensus about standardized therapeutic regimen for metastatic gastric cancer has been achieved yet, so that a variety of alternative, multidisciplinary therapies have been recommended by clinical practice guidelines, including RFA,^37^ transarterial chemoembolization (TACE),^64^ microwave coagulation therapy (MCT),^53^ adjuvant chemotherapy, molecular targeted therapy, or palliative supportive care.^65–67^ In particular, RFA, MCT, and TACE could additionally be used in the case of isolated metastasis in either half of the liver, given the absence of extra-hepatic disease.^68,69^ For example, in some groups of patients treated with RFA, survival rates resulted similar to those reported in the best surgical series.^12,70,71^

## CONCLUSION

In summary, despite the possible presence of a selection bias that included in the treatment group only patients with a more acceptable oncologic burden compared to that of the nonsurgical treatment group, the meta-analysis of multivariate data still shows a survival advantage of the local treatment of hepatic metastases. At this point, an international prospective study would be needed to clearly assess the feasibility and complications of local treatment of gastric cancer liver metastases. Then, it will possible to plan specific randomized clinical trials to fully understand the effectiveness of local treatment of gastric cancer liver metastases. Furthermore, due to the current lack of information in the published multivariate analysis it is important for future studies that the authors present a multivariate survival analysis reporting at least adjusted HR and CI. Meanwhile, our results suggest that surgical approach in case of hepatic metastases from gastric cancer should always be considered after conducting a multidisciplinary discussion, a proper patient selection, and given the absence of additional secondary tumors or extra-hepatic metastases.
